# Supplementary material for: End Sequence Analysis Toolkit (ESAT) expands the extractable information from single-cell RNA-seq data
Source: Genome Res. 2016 Oct;26(10):1397–410. doi: 10.1101/gr.207902.116 (PMC5052061; doi:10.1101/gr.207902.116)
Supplement: Supplemental Material [file supp_gr.207902.116_Supp_Materials.tar.gz › ESAT-esat_v0.1/doc/html/classumms_1_1core_1_1model_1_1score_1_1_abstract_list_score-members.html]

ESAT: Member List


ESAT


- Main Page
- Related Pages
- Packages
- Classes
- Files

- Class List
- Class Index
- Class Hierarchy
- Class Members

All Classes Namespaces Files Functions Variables Enumerator Pages

- umms
- core
- model
- score
- AbstractListScore

umms.core.model.score.AbstractListScore Member List

This is the complete list of members for umms.core.model.score.AbstractListScore, including all inherited members.

|  |  |  |
| --- | --- | --- |
| AbstractListScore(Annotation a) | umms.core.model.score.AbstractListScore |  |
| AbstractWindowScore(Annotation t) | umms.core.model.score.WindowScore.AbstractWindowScore |  |
| annotation | umms.core.model.score.WindowScore.AbstractWindowScore | protected |
| DEFAULT\_VALUE | umms.core.model.score.AbstractListScore | static |
| getAnnotation() | umms.core.model.score.WindowScore.AbstractWindowScore |  |
| getCount() | umms.core.model.score.AbstractListScore |  |
| getMean() | umms.core.model.score.AbstractListScore |  |
| getMedian() | umms.core.model.score.AbstractListScore |  |
| getScore() | umms.core.model.score.AbstractListScore |  |
| getScores() | umms.core.model.score.AbstractListScore |  |
| getStandardDeviation() | umms.core.model.score.AbstractListScore |  |
| getSum() | umms.core.model.score.AbstractListScore |  |
| setScores(List< Double > scores) | umms.core.model.score.AbstractListScore |  |
| toString() | umms.core.model.score.AbstractListScore |  |


---

Generated on Fri Aug 15 2014 15:11:11 for ESAT by  

 1.8.7
